# The Role of β-Cyclodextrin in the Textile Industry—Review

**DOI:** 10.3390/molecules25163624

**Published:** 2020-08-09

**Authors:** Fabricio Maestá Bezerra, Manuel José Lis, Helen Beraldo Firmino, Joyce Gabriella Dias da Silva, Rita de Cassia Siqueira Curto Valle, José Alexandre Borges Valle, Fabio Alexandre Pereira Scacchetti, André Luiz Tessaro

**Affiliations:** 1 Textile Engineering (COENT), Universidade Tecnológica Federal do Paraná (UTFPR), Apucarana 86812-460, Paraná, Brazil; fabiosachetti@utfpr.edu.br; 2 INTEXTER-UPC, Terrassa, 0822 Barcelona, Spain; 3 Postgraduate Program in Materials Science & Engineering (PPGCEM), Universidade Tecnológica Federal do Paraná (UTFPR), Apucarana 86812-460, Paraná, Brazil; helenfirmino@yahoo.com; 4 Postgraduate Program in Environmental Engineering (PPGEA), Universidade Tecnológica Federal do Paraná (UTFPR), Apucarana 86812-460, Paraná, Brazil; g.joycedias@gmail.com; 5 Department of Textile Engineering, Universidade Federal de Santa Catarina (UFSC), Blumenau 89036-002, Santa Catarina, Brazil; rita.valle@ufsc.br (R.d.C.S.C.V.); alexandre.valle@ufsc.br (J.A.B.V.); 6 Chemistry graduation (COLIQ), Universidade Tecnológica Federal do Paraná (UTFPR), Apucarana 86812-460, Paraná, Brazil; andretessaro@utfpr.edu.br

**Keywords:** cyclodextrin, dyeing, textile finishing, textile wastewater

## Abstract

β-Cyclodextrin (β-CD) is an oligosaccharide composed of seven units of D-(+)-glucopyranose joined by α-1,4 bonds, which is obtained from starch. Its singular trunk conical shape organization, with a well-defined cavity, provides an adequate environment for several types of molecules to be included. Complexation changes the properties of the guest molecules and can increase their stability and bioavailability, protecting against degradation, and reducing their volatility. Thanks to its versatility, biocompatibility, and biodegradability, β-CD is widespread in many research and industrial applications. In this review, we summarize the role of β-CD and its derivatives in the textile industry. First, we present some general physicochemical characteristics, followed by its application in the areas of dyeing, finishing, and wastewater treatment. The review covers the role of β-CD as an auxiliary agent in dyeing, and as a matrix for dye adsorption until chemical modifications are applied as a finishing agent. Finally, new perspectives about its use in textiles, such as in smart materials for microbial control, are presented.

## 1. Introduction

Since the first publication on cyclodextrins (CDs) in 1891, and the first patent in 1953, many technological advances have occurred, and the application of CDs has expanded [1]. According to Szejtli [2], over the years, CDs have been used in many diverse areas, and are identified, among all the receptor molecules, as the most important. 

This scenario is no different in the textile sector, which constantly seeks technological innovation, especially in the dyeing, finishing, and water treatment sectors. With the market and consumers increasingly demanding environmental improvements, the development of new features combined with green processes has become a constant challenge [3]. Among the various materials that can be used for this purpose CDs stand out; they are oligosaccharides made up of glucose units that are organized in a conical trunk shape, providing a well-defined cavity for the formation of host–guest complexes with a series of molecules [4]. This versatility allows complexation with drugs, dyes, insecticides, essential oils, cosmetics, and other compounds [5,6,7,8,9], allowing this class of molecules to assume a leading role in the textile industry.

**Figure 1:**
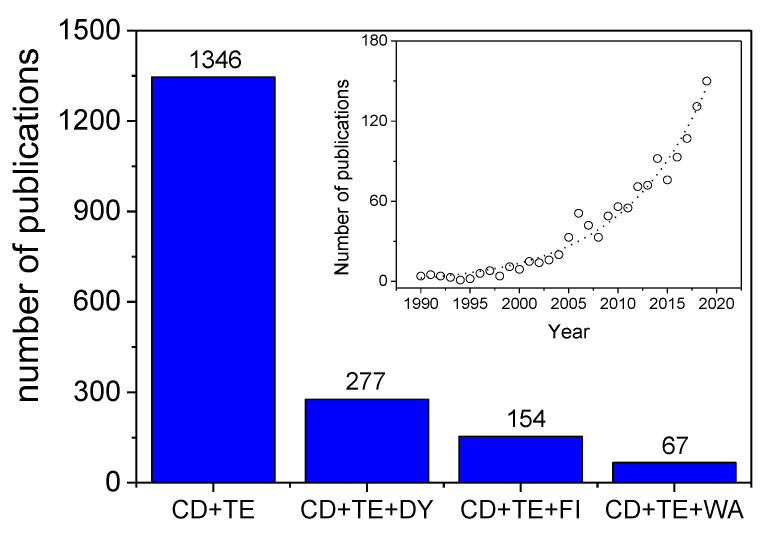
Number of publications available from SCOPUS when cyclodextrin (CD); textile (TE); dyeing (DY); textile finishing (FI); and textile wastewater (WA) are selected as keywords.

For the period from 1948 until today, since the term cyclodextrin started to be used as a research topic, 46,989 research papers have been reported by SCOPUS, and this number is continually increasing, as shown in Figure 1. This growth became significant in 1996, when the terms cyclodextrin and textile were combined and used as a research topic. These data were downloaded on 6 June 2020. 

Furthermore, due to the presence of numerous hydroxyl groups either in the interior or exterior, CDs are susceptible to the addition of new functional groups, which may yield new properties and functionalities. Additionally, CDs have a set of outstanding characteristics, such as high biodegradability, high biocompatibility, and approval by Food and Drug and Administration (FDA), which makes them human and environmental-friendly [10]. Therefore, this review presents an overview of the use of cyclodextrins, especially beta CD and its derivatives, in the textile field. Although some general physicochemical characteristics are presented, the scope of the work is focused on the application of CDs in the areas of dyeing, finishing, and wastewater treatment.

## 2. General Characteristics of Cyclodextrins

Initially known as Schardinger dextrins [11], the widespread use of CDs as hosts in supramolecular chemistry is relatively recent. Because they are natural products, CDs are biocompatible and accepted in biological applications; therefore, there is a growing interest in them both scientifically and industrially [12]. The optimization of methods for obtaining and applying CDs is, as a result, constantly evolving [7].

CDs are obtained through the enzymatic degradation of potatoes, corn and rice starch, which gives a mixture of linear, branched, or cyclic dextrins [13]. Initially, the cyclization reaction of the starch glucopyranose linear chains occurs by the enzyme cyclomaltodextrin-glucanotransferase (CGTase) [14], produced for example by *Bacilus firmus*. This step results in a mixture of α-CD, β-CD and γ-CD, composed of six, seven and eight units of D-(+)-glucopyranose, respectively, joined by α-1,4 bonds [15].

Subsequently, the separation and purification of these three CDs are required [5,16]. Among the methods used for this purpose, the most simple and widely used to isolate α-, β- and γ-CD is selective precipitation, forming inclusion complexes with an appropriate guest molecule—for example, α, β and γ-CD crystallize with 1-decanol, toluene, and cyclohexadec-8-en-1-one, respectively [7]. However, separation has a relatively high cost, making the entire synthesis process expensive. Fortunately, over time, research intertwined with the production of CGTase has evolved and allowed the isolation of α, β and γ-CGTase, increasing yield and consequently decreasing the production costs of the CDs [7].

As a structural consequence of the glucose units connecting through α-1,4 bonds, CDs occur as conical trunk shaped structures which are capable of solubilizing and encapsulating hydrophobic molecules in an aqueous environment [4,17,18].

The structure of CDs consists of primary hydroxyl groups (C6) located at the end of the rings, and secondary hydroxyls (C2 and C3) located at the outer edge of the rings. Ether type oxygen and polar hydrogen groups (C3 and C5) are present inside the trunk of the CDs. While the external hydroxyls are responsible for the relative solubility of CDs in water and micro-heterogeneous environments, the glycosidic oxygen bridges and, consequently, their pairs of non-binding electrons facing the interior of the cavity give this region, in addition to its Lewis basic character, hydrophobicity [18,19], making it capable of complexing nonpolar molecules [20]. The chirality caused by the five chiral carbons of the D-glucose unit associated with the rigidity of the macrocycle due to the intramolecular hydrogen interactions between the 2- and 3-hydroxyl groups are fundamental characteristics of the chemistry of CDs. Table 1 lists the physical properties of natural CDs.

The data presented in Table 1 indicate an apparent regularity in some properties, however, irregularities have been observed regarding the degradation temperature and solubility. Szejtli [2] has suggested that the lower solubility of β-CD is associated with intramolecular hydrogen bonds occurring at the edge. Although it has the lowest solubility, β-CD and its derivatives are the most used due to factors such as simplicity in obtaining it, lower price, reduced sensitivity and irritability to skin, and the absence of mutagenic effects [22]. 

The limitations imposed by the reduced solubility combined with the expressive attractiveness cause CD derivatives to be synthesized industrially. The CD derivatives that are most industrially produced include methylated β-CD, heptakis (2,6-dimethyl)-β-CD, heptakis (2,3,6-trimethyl)-β-CD, hydroxypropyl-β-CD, peracetylated β-CD, sulfobutyl ether-CD, and sulfated CD [10,23,24]. All have greater solubility in water compared to natural CDs, expanding the spectrum of applications in the controlled release of drugs, increasing blood solubility and bioavailability of medicines and textile deodorants, and assisting in polymerization [5,21,25]. Studies on their toxicology, mutagenicity, teratogenicity, and carcinogenicity have been carried out, and have shown negative results [7,26,27]. CDs also have hemolytic activity in vitro; β-CD has the highest and γ-CD the lowest activity [27,28].

The industrial applications of CDs are very diverse; they have been used in the pharmaceutical industry, in agriculture, in the textile area, in food technology, in chemical and biological analysis, in environmental protection, and in cosmetics [6,9,29,30]. 

CDs play a significant role in the textile industry, as they can be used to remove surfactants from washed textile materials [31], as leveling agents in dyeing [32,33,34], in textile finishing [35,36,37,38,39], and in wastewater treatment [40,41,42,43].

### Host-Guest Complex Formation

A striking feature of CDs is that they can form inclusion complexes with a variety of organic or inorganic compounds, allowing for the subsequent controlled release of these active compounds [44,45]. The fundamental factor for the guest molecule to be able to form a complex with the CD (host molecule) is its suitability within the cavity, which can be integral or partial [38,46,47]. Note that in the complexation, no covalent chemical bonds occur between the guest-molecule, nor is the compound closed within the macromolecular structure, which makes this type of complexation unique in terms of behavior as a modeler for the release of compounds [21,37].

Thus, the appropriate choice of the CD to be used for the possible formation of a complex is of great value. For small molecules, it is easier to form stable complexes with α-CD and β-CD, due to the compatibility of the volume of the guest molecule and the size of the CD cavity (Table 1). In the case of γ-CD, if the guest molecule is too small, the fit becomes unfavorable due to the much larger size of the cavity [19,48].

The general trend of CD complexation thermodynamics can be understood based on the concept of size; that is, by the analysis of the size and shape of the included molecule, and critical factors in the van der Waals interactions. Therefore, due to the fact that the cavity diameter of α-CD is much smaller than that of β-CD, and because the van der Waals forces are dependent on the distance between the molecules, it is expected that the forces induced by the complexation of extended chain molecules will be greater for α-CD than for β-CD [49].

As long as the fit-size requirements are satisfied, a number of other factors contribute to the complexation thermodynamics of the guest molecule in CDs. Considering only the aqueous environment, the following can be mentioned: (i) the entry of the hydrophobic portion of the guest-molecule into the CD cavity, (ii) the dehydration of the guest molecule and the exclusion of water molecules from the interior of the cavity, (iii) interactions of the hydrogen bonds between specific groups of the guest molecule and the OH of the receptor, and (iv) changes in conformation and/or stress reduction [49]. Although the preference for inclusion is of the hydrophobic portion (i), since charged species and hydrophilic groups are located in the bulk, certain groups with a hydrophilic character, such as phenolic OH, penetrate the cavity [50] and interact (iii) with the receptor. 

According to Venturi et al. [48], after complexation in an aqueous environment, the new chemical environment experienced by the guest molecule causes changes in its chemical reactivity. In numerous cases, an increase in stability, reduction in volatility, stabilization against light, heat and oxidation [47,51], solubility of the guest molecule in the solution, increase in the speed of dissolution [52,53], and bioavailability [54,55] were observed. However, depending on the experimental condition and type of CD, the inclusion can be deleterious for the guest, for example, enhancing the chlorpromazine photodegradation as observed by Wang et al. [56].

In terms of the stoichiometry of the inclusion complex, the four most common types of complexes are considered in CDs: guest molecules with a 1:1, 1:2, 2:1 and 2:2 ratio [57]. However, Pinho et al. [10] point out that the most common cases of complexation are 1:1 and 1:2. These configurations are dependent on the size and structural aspect of the guest-molecule in relation to the cavity of the CDs, allowing the formation of stable inclusion complexes [58].

However, Rama et al. [59] highlight that the chemical composition of the guest molecule, as well as its solubility, ionization state, and molecular mass, in addition to the conditions of the medium, such as the pH, temperature, solvent used, and other parameters, influence the process. The choice of the appropriate medium, working temperature, pH, and other factors will determine the best conditions for the interaction between the CD and the guest molecule [60,61]. Voncina et al. [62] highlight that an increase in temperature in the dyeing of polyacrylonitrile with cationic dyes using β-CD improves complexation, which reaffirms the importance of these parameters in the process. Other determining factors are related to the type of cyclodextrin used and the method of preparation: physical mixing [63], kneading [64], atomization [65], lyophilization [66], or coprecipitation [67].

**Figure 2:**
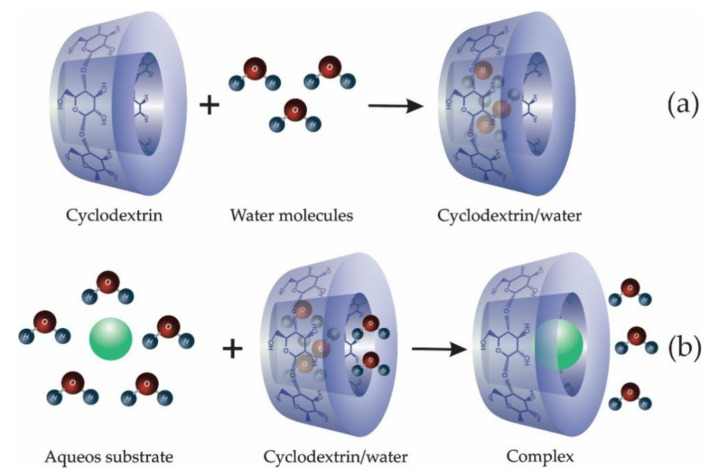
Complexation system. (**a**) Inclusion of water molecules in the cyclodextrin cavity; (**b**) complexation mechanism of the guest molecule in aqueous medium.

The mechanism of the formation of inclusion complexes can be divided into several steps; an illustration is shown in Figure 2. In the complexation of a substance in aqueous solution, the ends of the isolated CD cavity are opened in such a way that the guest molecule can enter the CD ring from both sides. There is, in principle, the absence of the guest molecule, and the slightly non-polar cavity, which acts as a host, is occupied by water molecules that are energetically unfavorable, as seen in Figure 2a. Given the nature of the polar–non-polar interaction, they can be easily replaced by a guest molecule that is less polar than water [14,68] (Figure 2b).

The molecules interact with each other as they are influenced by forces arising from the characteristics that are specific to each substance. Then, a complex phenomenon of molecular interaction occurs, since each interaction corresponds to a set of distinct forces [48]. Complexation is characterized by the absence of formation and the breaking of covalent bonds [69]. The driving force of the process is the increase in the entropy caused by the exit of water molecules present in the cavity and their consequent freedom [21]. Other forces also contribute to the maintenance of the complex, such as the release of the ring tension (especially for α-cyclodextrin), van der Waals interactions, hydrogen bonds, and changes in the surface tension of the solvent used as a medium for complexation [37,70]. 

## 3. Application of Cyclodextrins in the Textile Area

The wide range of applications of CDs has attracted the attention of many industries; however, according to Venturini et al. [48], initiatives for the industrial application of CDs have not been widely considered for three reasons: their scarce quantity and high prices, incomplete toxicological studies, and the fact that the knowledge obtained about CDs was not yet broad enough to envision their use in industry. The 1970s and 1980s were of fundamental importance for the diffusion of CDs in industry. Several studies have been successful in the production of CDs and their derivatives, and reliable tests have reduced doubts about their toxicity [2]. Their introduction into textile-related studies took on increasing relevance from the 1990s, according to SCOPUS data.

Bhaskara-Amrit et al. [31] emphasize that CDs have a very important role in textile processing and innovation; their use provides immediate opportunities for the development of environmentally friendly products and eco-textiles, in addition to having great potential in various applications. Cyclodextrins can be applied in the areas of spinning [71], pretreatment [72], dyeing [62,68,73], finishing [44,74,75,76,77,78], and dye removal [40,79,80,81], with dyeing, finishing, and water treatment being the most applicable in the textile area registered so far.

### 3.1. Dyeing Process

**Figure 3:**
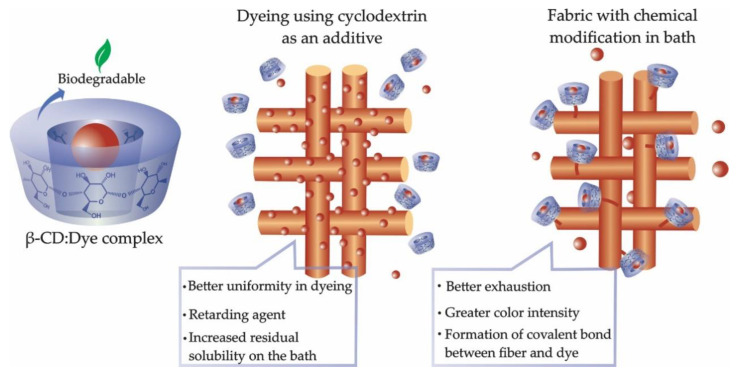
Use of cyclodextrins in dyeing.

The use of cyclodextrins in the dyeing process can include their use as a dyeing aid, forming a complex with the dye [33,82], or as a chemical modification of the surface [83,84]. Figure 3 shows these two processes.

CDs can form a variety of inclusion complexes with textile dyes (Table 2), and this inclusion changes their properties, directly influencing the quality of the dyeing. Therefore, cyclodextrins can be used as auxiliaries in the dyeing process.

#### 3.1.1. Cyclodextrin as an Auxiliary Agent in Dyeing

The introduction of new auxiliaries in the textile industry is feasible when they are used in low concentrations, are biodegradable, and do not affect the quality of the effluent. In addition to being biodegradable, CDs do not cause problems in textile effluents, and they improve the biodegradability of many toxic organic substances [17,68]. Their use is due to their formation of complexes with dyes, and they can be used to improve the uniformity of dyes and washing processes [31,82,86]. However, for the cyclodextrin to act as an auxiliary, the formation of the CD:dye complex is essential; if it is not formed, there will be no improvement in the quality of the dyeing [75,95].

In the dyeing of polyester fibers, dyes of the dispersed type are used for the coloring of the substrate [100]. These dyes are poorly soluble in water and generally require the use of surfactants [73], which are products from non-renewable sources that cause environmental problems and must be replaced to reduce damage [85]. Therefore, cyclodextrins are an alternative to these products that can maintain the quality of the coloring of the textile article [68]. 

Carpignano et al. [73] conducted studies on the application of β-CD with dispersed type dyes and polyester, and stated that the presence of β-CD positively affects color uniformity, intensity, and bath exhaustion when compared to dyeing using commercial surfactants. The insertion of cyclodextrins into the dyeing process decreases the amount of free dye molecules [33], causing the dyeing rate to decrease and favoring leveling [91]. This is due to the fact that the complex (CD:dye) has a molar mass greater than that of free dye, hindering its diffusion into the fiber, thus favoring the dye delay mechanism, which causes better leveling [101].

Another important synthetic fiber in the textile area is polyamide. This fiber presents, at the ends of its chains, carboxylic and amine groups, which gives it a substantivity for several classes of anionic dyes [102]. Commercially, acid dye is the most used due to the dye–fiber interaction in the acid medium, the leveling results, and the achieved colors [103]. Dispersed dye is seldom used due to its low adsorption and the possibility of a barre effect; therefore, in order to be able to use dispersed dyes for the dyeing of polyamide, it is necessary to improve the leveling and adsorption of this dye by the fiber. This can be achieved when using cyclodextrins as an auxiliary agent in dyeing [32].

Ferreira et al. [87] studied the dyeing of a polyamide 6 microfiber using dispersed dye complexed with cyclodextrins, and found that the complex changes the dyeing kinetics, improving its distribution in the fiber. There are also changes related to the thermodynamics of dyeing, since the dyeing also proved to be more intense, with greater adsorption of the dye by the fiber related to the increase in the dispersibility of the dye in the aqueous phase [88]. Similar results were found by Savarino et al. [89] when they dyed polyamide 6 with dispersed dyes, showing changes in the kinetic and thermodynamic phases of the dyeing. This indicates that cyclodextrins can replace additives from non-renewable sources and improve the dyeing and the effluent generated.

With regards to natural fibers, cotton is one of the most important textile fibers [104] and, in its dyeing, the use of reactive and direct dyes stands out. Reactive dye has structure groups that covalently bond with the fiber, improving the solidity; however, they have low affinity, requiring high amounts of electrolytes for good dyeing to occur [105]. Direct dye, on the other hand, has a high affinity for the cotton fiber [106] and it is often necessary to use retarding agents, such as alkaline salts, to prevent stains on the fabric and thus achieve better leveling [107].

The use of cyclodextrins can help to solve these dyeing problems. Parlat et al. [91] dyed cotton with reactive dye using cyclodextrins as an auxiliary. In this case, as a result of the complexation of the reactive dye, there was a good diffusion of the dye into the fiber, increasing its uniformity and color intensity.

In the works of Cireli et al. [82], the insertion of CDs occurred in the process of dyeing cotton with direct dye. The CDs acted as a retarding agent, forming complexes with the dye molecules, causing the dyeing speed to decrease, which improved the leveling.

Other works performed dyeing using β-CD, such as those of Voncina et al. [62], who dyed polyacrylonitrile with cationic dye and observed an improvement in color intensity and exhaustion when compared to the use of quaternary ammonium. Shibusawa et al. [92], who dyed cellulose acetate with dispersed dye, found that the complex formed between CD: dye changed the speed at which the chemical balance of the process was achieved, making it slower.

In general, cyclodextrins inserted as an auxiliary affect both the properties of the dyes and the dyeing kinetics, allowing improvements in exhaustion, uniformity, and in the quality of the effluent water. However, it is worth mentioning that this is only achieved when inclusion of the dye in the cavity of the CD is achieved.

#### 3.1.2. Dyeing Chemical Modification

Some textile fibers present difficulty in dyeing due to the terminal groups present in their chains, causing some dyes to fail to create interactions, as is the case with polypropylene fibers [99] and vinylon fibers [95]. Other fibers present selectivity for dyes, such as cotton, which is not dyed by acidic and dispersed dyes [108]. However, promoting the modification of the surface of these fibers can cause new possibilities for the interactions between the dye and the fibers [109].

Cyclodextrins are polymers that can cause this chemical modification through incorporation into the fiber [99]. This incorporation can be seen as a pre-treatment for the dyeing or as a finishing, depending on the actions taken after modification. In this section, only the modifications for dyeing will be addressed and, in the next, finishing will be explored.

With cyclodextrins incorporated into the fabric, new groups and pores through which the dyes can fix become available. One fiber that presents difficulty in dyeing is cellulose acetate fiber, due to its compact structure, low content of polar groups, and hydrophobicity [110]. These factors make it difficult for dyes to diffuse in the fiber. To obtain better results in the dyeing process, Raslan et al. [75] treated the cellulose acetate fabric (38.5% acetyl) with monochlorotriazinyl-β-cyclodextrin (MCT-β-CD) using the padding technique to improve its dyeability. As a result, they were able to perform dyeing at a low temperature, improving the color intensity, and they also increased the diffusion of the dye within the fiber by about 70%.

In the case of polyester fibers, some authors have performed the process of acetylation [83] or coating [97] to modify the surface with CDs. This results in an improvement in the solidity of the dyeing [98], in addition to the possibility of dyeing with other classes of dyes. Zhang et al. [97], after performing the modification of polyester fiber, dyed this fabric with cationic dye. The fabric showed a gain in hydrophilicity, a reduction in the dyeing temperature to 70 °C, and interaction between the crosslinking carboxylate groups and the cationic dye, in addition to its complexation by the CDs.

Another work that used the modification of the polyester surface with cyclodextrins was carried out by Chen et al. [94]. In this work, the modification enabled a 47% increase in the color intensity in the stamping process, a fact associated with the greater sharpness and depth achieved by the dyes. In addition, the CDs, when chemically bonded to the fabric, can act as an anti-migration agent, because during the drying or curing of polyester fabrics dispersed dyes tend to migrate to the fabric surface and the CDs act as a dye sequestrant, consequently preventing this dyeing defect [83].

In the case of the modification of cotton fiber with cyclodextrins, several routes are possible, but the most used is esterification using citric acid or 1,2,3,4-butane tetra-carboxylic acid (BTCA) [111] as crosslinking. These changes will be covered in more detail in the next section. Rehan et al. [96] carried out the modification of cotton fiber with CDs and citric acid to perform dyeing with acid dye. These dyes present low affinity for the dyeing of cellulosic fiber [108]. After the modification, the authors realized that the dye was adsorbed by the cyclodextrins, which allowed the dyeing to achieve satisfactory solidity.

In general, the modification of the fiber surface through the insertion of cyclodextrins increases the adsorption of dye and allows a greater variability of dye classes in fibers that have no affinity, often achieving better color standards in multi-fiber items [97,98] and improving the efficiency of the dyeing process for fibers that require greater use of auxiliaries to achieve the proper color standard.

### 3.2. Textile Finishing

**Figure 4:**
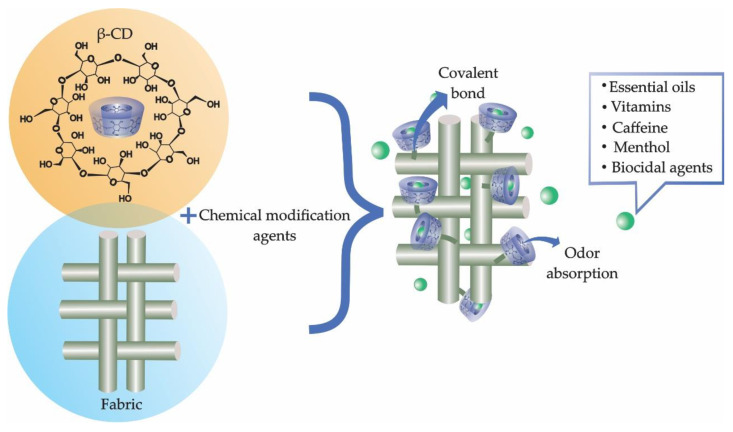
Use of cyclodextrins in textile finishing.

In the area of textile finishing, cyclodextrins can have many applications; they are able to absorb unpleasant odors, and act as an encapsulation agent for essential oils [38,76,78,112,113], vitamins [114], hormones [77] and biocides [6,115] in order to preserve compounds and/or control their release, as shown in Figure 4. The loading of active ingredients allows the incorporation of specific and desired functions into textile materials, which may act differently under particular uses, such as in medicine [116], cosmetics [117], and engineering [118].

In numerous cases, the complexation of active ingredients by CDs improves their physicochemical properties, controls their release, maintains bioavailability, increases shelf life, provides storage conditions, reduces environmental toxicity, increases chemical stability, protects against oxidation, and favors resistance to repeated washing [6,7,114,119,120].

In order to make it possible to incorporate these active molecules into the textile substrate, there is a need to fix the CDs in the fiber. Several methods have been proposed for the permanent fixation of CDs into textile fibers, and in some cases, there is a need for a first step—the modification of the cyclodextrins—so that they can be incorporated into the fabric. The selection of the best method for fixing CDs into a textile substrate depends on different factors, the main ones being reactivity of the cyclodextrins to the final application, and the type of fiber [23,121].

#### 3.2.1. Preparation of Cyclodextrins

**Figure 5:**
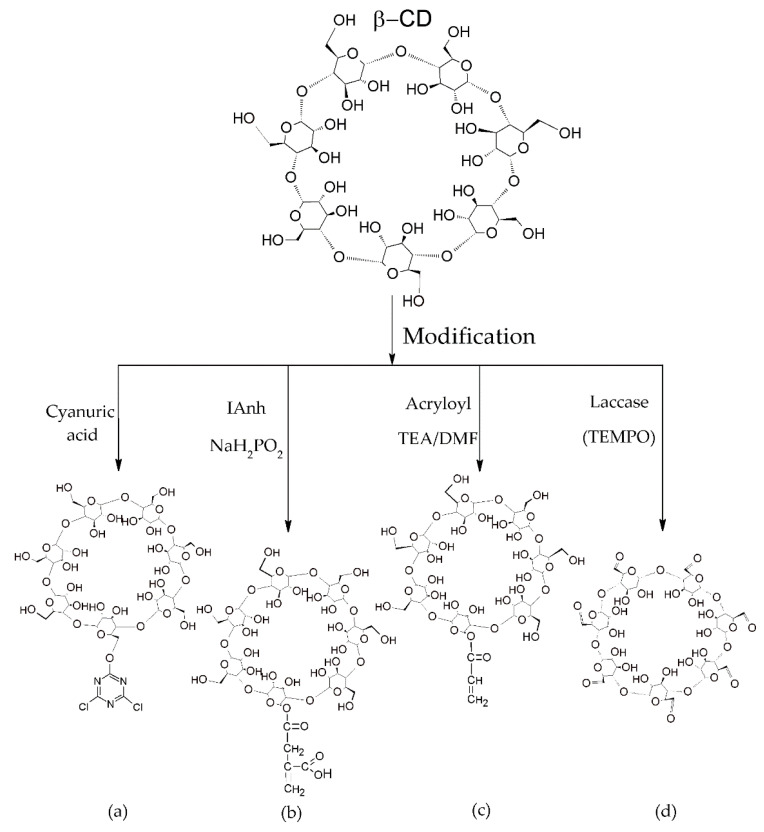
Modification of β-CD by: (**a**) cyanuric acid; (**b**) itaconic anhydride (IAnh); (**c**) acryloyl chloride and (**d**) laccase/2,2,6,6-tetramethylpiperidine-1-oxyl (TEMPO) enzyme.

Cyclodextrins are capable of forming complexes with a wide range of molecules, but they cannot form a direct covalent bond with textile materials; therefore, some cyclodextrin derivatives have been synthesized with reactive groups to allow them to chemically bond to various substrates [122], as shown in Figure 5. 

One of the most common reactive derivatives of cyclodextrins is MCT-β-CD, as seen in Figure 5a, synthesized through the reaction between cyanuric chloride and β-cyclodextrin [123]. MCT-β-CD is the most interesting derivative used on cellulosic substrates due to the simple bonding process in relatively mild conditions. The monochlorotriazine groups incorporated into the CDs react by a nucleophilic substitution mechanism, and form covalent bonds with the hydroxyl groups of the cellulose [124]. Another product that can be synthesized from MCT-β-CD is the cyclodextrin polymer (6^A^-O-triazine-β-cyclodextrin), produced by polycondensation using β-CD and cyanuric chloride [125].

Formation occurs due to nucleophilic substitution, in which the hydroxyl groups of the CDs react with the chlorine contained in the cyanuric chloride, and thus form the β-CD copolymer [125]. From the formation of this compound it is possible to create interactions with the hydroxyl groups present in the textile fibers; this occurs by substitution.

The modification of CDs can also be performed using itaconic acid (Figure 5b) containing carboxyl and vinyl groups. This bifunctional compound can be linked to the CDs via an esterification reaction, and its vinyl group can perform polymerization by free radicals [5,122]. Itaconic anhydride is obtained from itaconic acid at 110 °C in the presence of sodium hypophosphite [122]. From the modification of the CDs, the end containing the itaconic anhydride is able to bond with the textile fibers through covalent reactions.

Another CD modification for incorporation in textiles can be carried out via a reaction with acryloyl derivative (Figure 5c). The CDs are dissolved in dimethylformamide (DMF), mixed by stirring with triethanolamine (TEA), and reacted with acryloyl chloride dissolved in DMF, forming an acryloyl ester derivative [126]. The compound has a vinyl group on the side chain that is able to react with hydroxyl groups, and can be incorporated into the fibers [23,124].

In addition to the reaction through the incorporation of new chemical groups into the CDs, to make them more reactive hydroxyl groups can be oxidized, as can be seen in Figure 5d. The hydroxyl groups in the polysaccharides can be oxidized by a laccase/2,2,6,6-tetramethylpiperidine-1-oxyl enzyme catalyzed to convert the hydroxyl groups of the CDs into aldehyde groups that are capable of reacting with the amino groups of polyamide, silk, and wool [127].

#### 3.2.2. Grafting of Cyclodextrins onto Textile Substrates

The most common procedure in the application of cyclodextrin into textiles is esterification, which can be done using modified cyclodextrins (Figure 5), or through a reaction using dimethylol urea [128], citric acid [111], BTCA [78,129], or other products.

Esterification can be defined as a nucleophilic substitution reaction of the acyl group catalyzed by a mineral acid, involving a carboxylic acid and an alcohol [130]. From there, a proton transitions from one oxygen to another, resulting in a second tetrahedral intermediate, and converts the -OH group into a leaving group, culminating in the loss of a proton that regenerates the acid catalyst, originating the ester [131].

**Figure 6:**
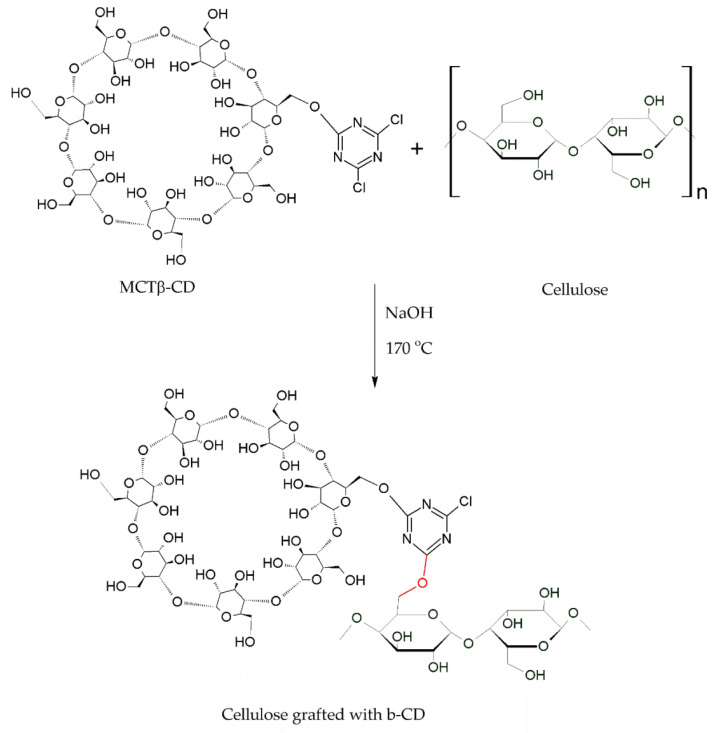
Nucleophilic substitution reaction of MCT-β-CD with cellulose.

Figure 6 shows the procedure for incorporating MCT-β-CD into cellulosic fiber. The interaction occurs due to the availability of the chlorine group present in MCT-β-CD and the hydroxyl group of cellulose, thus representing a second order nucleophilic substitution reaction [132].

MCT-β-CD is fixed on cellulosic fibers in alkaline conditions and, due to the covalent bond between the cellulosic chain and MCT-β-CD, the durability of β-CD in textile products is excellent [23,133]. 

Ibrahim et al. [134] also used MCT-β-CD for the functionalization of wool by a method of fixation in foularding. Due to the presence of -OH groups in the protein, it is also possible to perform nucleophilic substitution. As with polyamide fabrics and polyester/cotton blends, this β-CD derivative has also been grafted, making the fabric antibacterial and a receptor for drugs and essential oils, in addition to improving thermal stability and dyeability [128,130]. 

The MCT compound was also used to make polyester a functional fabric, made from alkaline hydrolysis, which created reactive hydroxyl groups on the surface of the polyester fibers able to react with MCT-β-CD covalently [39]. From the interaction with the cyclodextrins, the modified polyester can adsorb bioactive molecules [112].

Cyclodextrin compounds treated with itaconic anhydride can bind to cellulosic and polyamide fibers. In the case of cellulosic fibers, the fabric must be treated with a mixture of nitric acid (1%) and cholic ammonium nitrate to generate free radicals and, after drying, the cotton is treated with a derivative of CD itaconate, which is able to covalently bond to cellulosic fibers [5,122].

In addition to the processes using modified cyclodextrins, esterification between cyclodextrins and textile fibers can be achieved. In this case, the esterification reaction requires a crosslinking agent such as citric acid, BTCA, or other polycarboxylic acids [135]. The disadvantage of using citric acid is the yellowing of the cellulosic fabric in the curing phase [136]. This process includes two steps; in the first, a cyclic anhydride is formed between two groups of adjacent carboxylic acids and, in the second, the esterification reaction occurs between the acid anhydrides previously formed and the hydroxyl groups of the macromolecules of the fiber and of the cyclodextrins, to form ester bonds [23].

**Figure 7:**
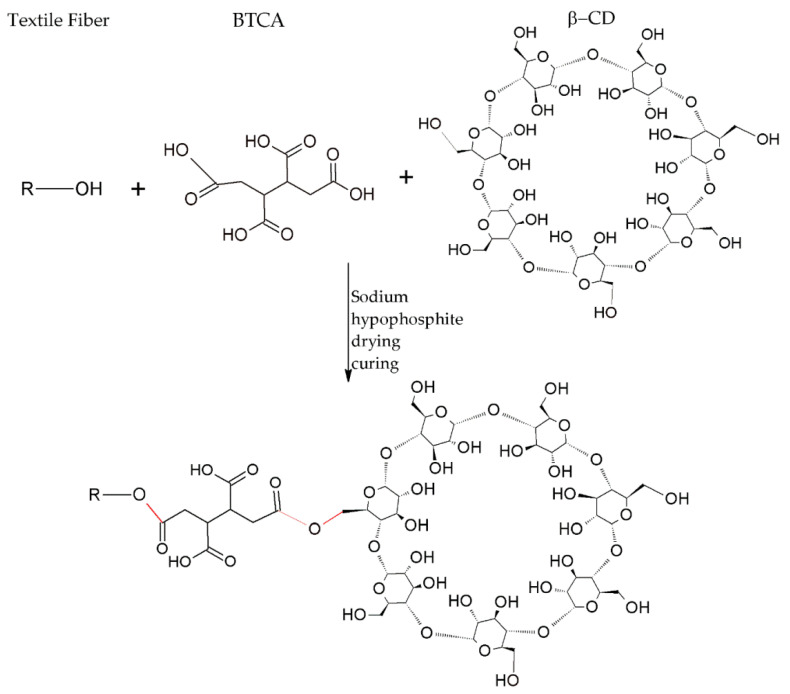
Direct connection of the β-CD to the textile fiber via crosslinking.

Figure 7 illustrates the bonding between CDs, through BTCA as a crosslinking agent, and -OH groups of fibers.

For the esterification reaction to occur, both sodium hypophosphite and the cure are used as catalysts [38]. The same process can be performed on other fibers that have -OH groups, such as cellulose, silk, polyamide, and wool [78].

Regarding the insertion of cyclodextrins into polyester fibers, they can be functionalized by forming a network of CDs that cover the fiber, forming a reticulated coating between β-CD and BTCA through a polyesterification reaction [38,137].

**Figure 8:**
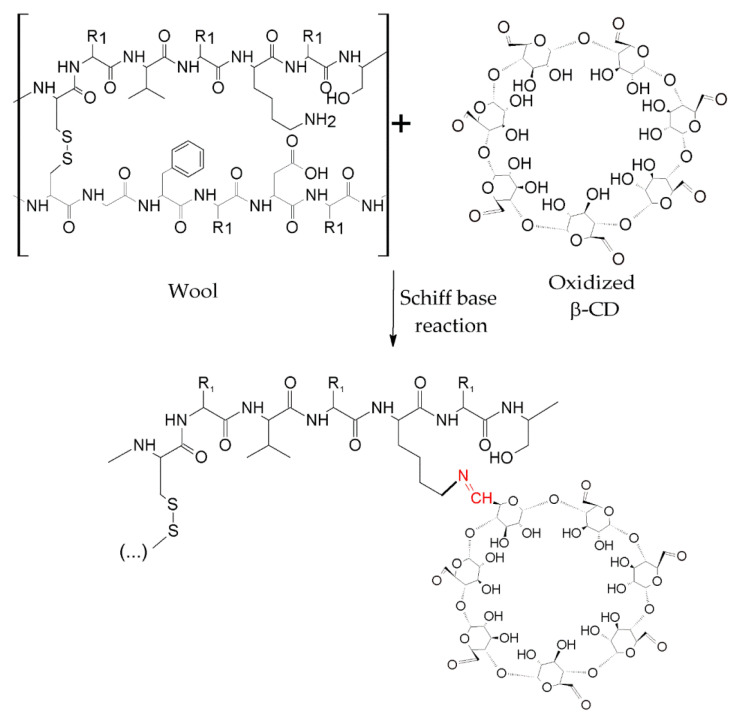
Functionalization of wool fibers with β-CD after oxidation.

As shown in Figure 5d, the hydroxyl groups of the CD can be oxidized by enzymes, converting them into aldehyde groups, which are able to react with the amine groups of the wool fibers through a Schiff-based reaction [127]. Figure 8 shows this reaction.

In this way, the application of CDs in fibrous polymers occurs. The substrate undergoes a change at the surface that can transform it, in the future, into functionalized fabrics after the complexation of the bioactive molecules by the CDs present on the surface of the materials.

Table 3 shows some studies that used cyclodextrin for the functionalization of finished textiles.

### 3.3. New Trends in Textile Finishes Using Cyclodextrins

**Figure 9:**
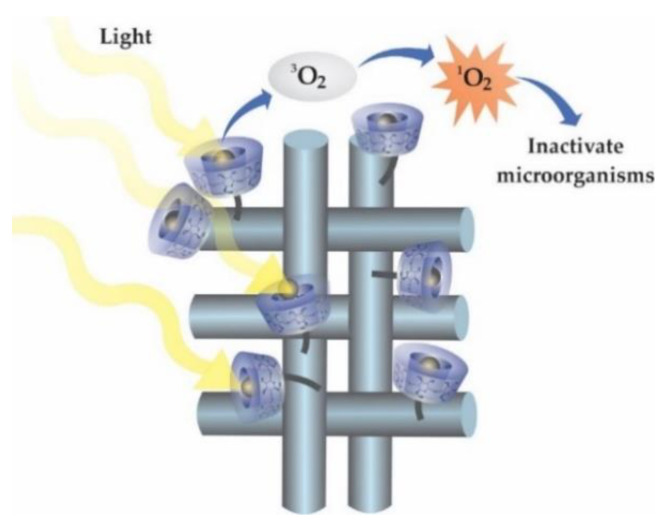
Finishing textiles with photodynamic potential.

The use of citric acid as a reticulating agent was also a strategy adopted by Castriciano et al. [154] to design polypropylene fabric finished with hydroxypropyl β-CD. After complexation with tetra-anionic 5,10,15,20-tetrakis(4-sulfonatophenyl)-21H,23H-porphine (TPPS), the textile device was evaluated as a biocidal agent via antimicrobial Photodynamic Therapy (aPDT)—an alternative treatment to overcome the drug resistance associated with the indiscriminate use of antibiotics. The base of aPDT is the irradiation of a photosensitizer (PS) in the presence of oxygen, to generate reactive oxygen species (ROS) which attack the microorganisms at the target site (Figure 9). The PP-CD/TPPS fabric, containing 0.022 ± 0.0019 mg cm^−2^ of the TPPS, was capable of photokilling 99.98% of Gram-positive S. aureus, with low adhesion of bacteria to the textile. The aPDT approach was also used by Yao et al. [155] to develop biocidal materials based on beta cyclodextrins modified with hyaluronic acid (HA) for coating purposes. After the inclusion of PS methylene blue (MB), HA-CD/MB was tested against S. aureus, eradicating 99% of the bacteria at 0.53 ± 0.06 μg cm^−2^. The use of aPDT in textile finishing may represent a new class of smart textiles with high anti-microorganism potential.

### 3.4. Cyclodextrins in Textile Effluent Treatment

Cyclodextrins, in addition to being used as additives for the dyeing process when seeking improvements in washing, color intensity, and leveling, and as a functionalization agent, can be used to remove dyes and auxiliaries present in industrial effluents [40,156]. In the wastewater from the dyeing process, the presence of several types of dyes, surfactants, and salts can be an issue [157]. The dyes used in dyeing are compounds that are stable to oxidizing agents and light, have a complex structure, are non-biodegradable, and are highly soluble in water. Therefore, they are difficult to remove and can easily enter the ecosystem, affecting flora and fauna [79,158,159,160,161,162].

Various technologies for the treatment of water from the textile industry are used, such as photocatalytic oxidation [163], electrochemical oxidation [164], membrane separation [165], coagulation/flocculation [166], ozonation [167], and biological treatment [168], among others; however, there are restrictions regarding these processes, due to the high energy consumption and sludge generation. Thus, the search for processes that can eliminate residues from the dyeing and finishing processes is essential to alleviate major environmental problems. Lin et al. [169] and Crini et al. [170] point out that, among the different treatment systems, adsorption should be highlighted. It has been increasingly used, mainly due to its adaptability, easy operation, and low cost.

**Figure 10:**
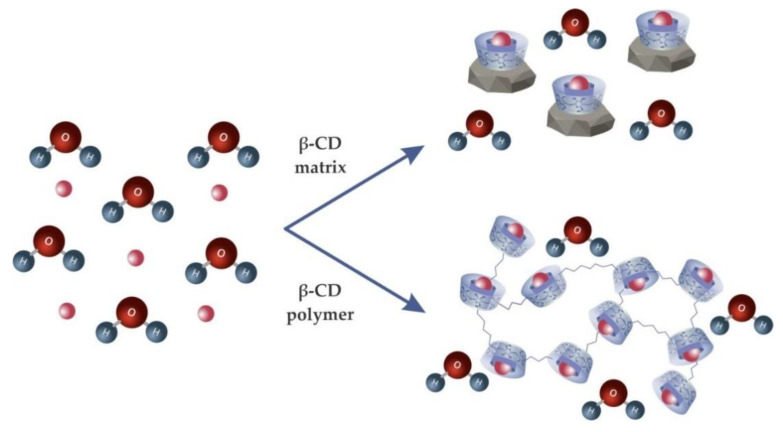
The role of β-CD as a dye adsorbent.

Among the adsorbents used, cyclodextrins are seen as a promising product [40] due to the high reactivity of the hydroxyl groups present in CDs for the adsorption process [171]. In addition, other advantages are related to its biodegradability, non-toxicity, availability [172], and the possibility of them interacting with the hydrophobic chain of surfactants, keeping them within its cavity [173]. In this way, they can form an insoluble CD:dye:surfactant system that can be removed from the water [79]. In general, Crini et al. [170] showed that the use of cyclodextrins as a dye adsorbent can be carried out by two methods, shown in Figure 10. 

In the first method, cyclodextrins are incorporated into an insoluble matrix (nanoparticles, composites, nanotubes, and others), while in the second, CDs form an insoluble polymer capable of adsorbing the dyes. Table 4 shows some studies that have used cyclodextrin for the removal of textile dyes.

#### 3.4.1. Cyclodextrin Matrix 

A material used for the adsorption of dyes present in effluents must have a high adsorption capacity, ease of regeneration, mechanical resistance, and ability to adsorb a variety of dyes [182]. This last characteristic is often neglected, with experiments being carried out on solutions that contain only one type of dye; however, Debnath et al. [161] showed that most wastewater contains a mixture of different dyes, which affect the behavior of the adsorption system differently to a single dye system.

Therefore, cyclodextrins, due to their well-defined structure, can guarantee high reactivity for the adsorption of various dyes [171], and this can be improved if they are inserted into other adsorbent materials. These hybrid materials have a high adsorption capacity due to large specific surfaces and pore volume [43]. 

One of the techniques used for the production of promising adsorbent materials is electrospinning [183]. Abd-Elhamid et al. [162] produced nanocomposites using polyacrylic as an incorporation matrix and graphene oxide and cyclodextrin as adsorbent materials. According to the author, the nanocomposite is easy to prepare and has a high sorption capacity and is easy to remove from water. The combination of cyclodextrins and graphene to obtain a hybrid adsorbent material was also used by Liu et al. [176], who, in this case, also used poly(acrylic acid). This nanocomposite showed efficiency in pollutant adsorption, water dispersibility due to the hydrophilicity of the polymer, ease of regeneration, and a small loss of adsorption capacity.

Cyclodextrins can also be used in the production of biosorbents, together with chitosan. Chitosan is a compound rich in hydroxyl and amino groups, which allows interactions with organic and inorganic compounds [184,185]. However, chitosan, if not modified, can dissolve in acidic solutions because of the protonation of amino acids, hindering the adsorption of dyes [186]. To avoid such a problem, chitosan can be crosslinked with carboxylic acids and, to improve this biosorbent, Zhao et al. [187] chemically incorporated cyclodextrins into chitosan by means of esterification using citric acid, obtaining a biosorbent with a high capacity for adsorption of reactive dyes from textile effluents.

Chen et al. [157] showed that some researchers have grafted β-CD into insoluble solids, such as zeolite, activated carbon, silica gel and magnetic materials, obtaining good adsorption results. These characteristics show that adsorbent materials with cyclodextrin incorporation have great potential for applications in wastewater treatment, due to their large amount of hydroxyl groups, hydrophobic cavity, and interactions with organic and inorganic compounds.

#### 3.4.2. Cyclodextrin Polymers

The synthesis of cyclodextrin polymers, especially those that are insoluble in water, has aroused growing interest given their applications in water treatment. Among the various methods of obtaining them, deprotonation stands out, in which the hydroxyl anion can be used in SN2 type polymerization reactions, direct dehydration in the presence of appropriate diodes and diacids, and condensation in the presence of a series of linkers [188]. In addition to polymerization, some studies have used β-CD for the development of organic-inorganic hybrid systems for the removal of dyes, such as magnetic CD polymers [40,189] and Halloysite−Cyclodextrin Nanosponges [190].

Crini et al. [81], using epichlorohydrin as a crosslinking agent for obtaining β-CD polymers, evaluated their efficiency in removing various dyes (acid blue (AB25), basic blue (BB3), reactive blue (RB19), dispersive blue (DB3) and direct red (DR81)). The capability to remove dyes by these polymers followed the order AB25 > RB19 > DB3 > DR81 >> BB3, with AB25 being close to 100% removed. The same author also prepared β-CD/carboxy methylcellulose polymers using the same crosslinking agent for the removal of Basic Blue 3, Basic Violet 3 and Basic Violet 10. Kinetic and equilibrium studies suggested that the process occurs by chemisorption, with an adsorptive capacity of 53.2, 42.4 and 35.8 mg of dye per gram of polymer for BV 10, BB 3 and BV 3, respectively [160]. 

Pellicer et al. [171] also used epichlorohydrin as a crosslinking agent to synthesize polymers of β-CD and HP-β-CD, which were used to remove the azo dye Direct Red 83:1. The adsorption capacity of the polymer synthesized from β-CD was approximately six times greater than that obtained using HP-β-CD. Ozmen and Yilmaz [191] used β-CD polymer, prepared using 4-4-methylene-bis-phenyldiisocyanate (MDI), to remove Congo red dye. The authors observed 80% removal after one hour of contact in solution at pH 5.8. The same authors, using MDI and hexamethylene diisocyanate (HMDI) with crosslinking agents, synthesized β-CD polymers and evaluated their adsorptive capacities against the azo dyes Evans Blue and Chicago Sky Blue. At pH 2, the polymers showed around 50% removal.

Jiang et al. [192] synthesized a new polymer of β-CD for the removal of methylene blue. The strategy used by the authors was the use of tetrafluoroterephtalonitrile (TFPN) as a crosslinking agent, which, after being hydrolyzed, generates sites of carboxylic acids that interact electrostatically with the MB at the appropriate pH. A maximum adsorption capacity of 672 mg/g of the polymer was observed and, even after four cycles of adsorption/desorption, the capacity of the material remained high. The same group of researchers used a similar strategy for the synthesis of β-CD polymers, however, the nitrile groups of TFPN were modified with ethanolamine. This strategy enabled the selective removal of MO in a mixture of MO and MB. The polymer also showed a high adsorptive capacity for MO (602 mg/g) and Congo red (1085 mg/g). Recently, the selective removal of the anionic dye Orange G in a mixture with methylene blue has also been carried out by modifying the TFPN nitriles to form amide groups [41]. An innovative strategy using molecularly imprinted polymers (MPI) from chitosan and β-CD was used for the selective separation of Remazol Red 3BS in a trichromatic mixture. This new polymer also showed a high adsorption capacity after four cycles of use [168].

Some multifunctional CD polymers have also been developed for the simultaneous removal of dyes and other contaminants (bisphenol and heavy metals). Zhou et al. [42] synthesized a polymer of β-CD using citric acid as a crosslinking agent, which, after esterification, was grafted with 2-dimethylamino ethyl methacrylate monomer (DMAEMA) for the polymerization reaction. This elegant strategy allows modulating of the zeta potential of the adsorbent with the pH, enabling its electrostatic interaction with anionic (MO) or cationic (MB) dye. Simultaneously, the material can adsorb Bisphenol A inside the CD, and its interaction with the CD is unchanged between pH 2 to 10. The adsorption capacity at equilibrium for Bisphenol A was 79.0 mg/g, while the adsorption capacity of MO and MB was, respectively, 165.8 and 335.5 mg/g. 

Zhao et al. [193] presented an elegant strategy for the treatment of industrial wastewater by means of a bifunctional adsorbent, consisting of a polymer of ethylene diamine tetra-acetic acid and β-CD (EDTA-β-CD). This bifunctional agent can simultaneously remove metals and dyes from wastewater, since β-CD has the ability to include dyes while EDTA becomes a site for metals. In experiments with binary systems containing Cu^2+^ and dyes (methylene blue, safranin O or crystal violet), the authors observed an increase in the adsorption capacity of the metal, but no significant change in the adsorption of the dyes, compared to experiments in systems with the isolated metal. The increase in the adsorption of the metal in binary Cu^2+^-dye systems was attributed to the presence of the complexed dye in the CD, which provides extra groups containing nitrogen that become new sites for the adsorption of metals.

Despite the efficiency of the CD-based polymer in removing dyes and other agents in the textile process, some important points should be highlighted. Most of the studies presented above still need to be applied at a high scale level (in a real industrial system). Another important issue that should be emphasized is the regenerability of the CDs, making the process more ecofriendly and viable, with a lower cost.

## 4. Final Considerations and Future Perspectives 

The increasing use of CDs in the textile industry is the result, among other factors, of the versatility of these cyclic molecules and the benefits of their use across the productive chain of this sector. Their unique ability to form an inclusion complex with a wide variety of molecules allows their use in several sectors. CDs are able to include dyes, repellents, insecticides, essential oils, caffeine, vitamins, drugs and surfactants, among other substances. Although they are used in the spinning and pretreatment areas, it is in the dyeing, finishing, and water treatment processes that β-CD and its derivatives have the greatest applicability.

The advantages of using CDs in dyeing include changes in bath exhaustion, color uniformity, less effluent treatment, dye savings, and the fact that they are biodegradable. They can be used as a dyeing aid, or as a surface modifying agent that absorbs more dye.

With regard to finishing, different types can be made with CDs, expanding the range of applications for these textiles and giving rise to a new class of materials called functional or intelligent textiles.

It is foreseeable that the use of CDs will continue to expand to keep up with the demands for differentiated products, and fill the gap that still exists in the literature around their application in the textile area, aiming at the optimization of the processes and viable results for industrial use.

This functionalization of CDs in substrates opens the door for the development of new products, such as medical textiles. With the new reality caused by the SARS-CoV-2 pandemic, the development of antiviral textiles is on the rise, and many of these new materials could be generated from technologies that use CDs. Furthermore, the transposition of new medical treatment technologies into textile materials from CDs is already a reality. An example is the use of β-CD for the development of textiles aiming at the photodynamic inactivation of microorganisms.

Finally, the capacity of CDs to adsorb and separate pollutants (dyes, metals, surfactants, etc.) from industrial waste is important with regards to environmentally sustainable industrial processes. In addition to adaptability and ease of operation, their biodegradability and lack of toxicity make CDs stand out in different areas.

Without a doubt, the use of CDs in basic and applied research around the development of new materials is fundamental, and should be the focus of many future studies seeking sustainable alternatives in the textile area.

## Figures and Tables

**Table 1:** Some physicochemical properties of cyclodextrins [2,21].

Properties	α-CD	β-CD	γ-CD
Empirical formula	C_36_H_60_O_30_	C_42_H_70_O_35_	C_48_H_80_O_40_
Molecular weight (g/mol)	972	1135	1297
Glucopyranose units	6	7	8
Cavity diameter (nm)	0.47–0.57	0.60–0.78	0.83–0.95
Internal cavity volume (nm^3^)	1740	2620	4720
Number of water molecules in the cavity	6	11	17
Aqueous solubility (g/L)	129.5	18.4	249.2
Temperature of degradation (°C)	278	298	267

**Table 2:** Examples of dyeing CD applications, as a textile aid and as a chemical modifier.

Application of Cyclodextrins	Fiber	Dye	Reference
Auxiliary agent	Polyester	Disperse	[73]
		Synthetic	[85]
		Disperse Orange 30, Disperse Red 167, Disperse Blue 79	[86]
		Methylene Blue	[84]
	Polyamide 6	Disperse Red 60	[87]
		Disperse	[88]
		Synthetic	[32,89,90]
	Nylon, polyester and cotton	Synthetic, reactive and disperse dye	[91]
	Cellulose Acetate	Azo disperse	[92]
	Polyacrylic	Basic Blue 4	[62]
	Cotton	Direct	[82]
	Wool	Natural (*Bixa orellana*)	[93]
Chemical modification	Polyester	Pigment inks carbon black, magenta, yellow and cyan	[94]
		Disperse Red 60, Disperse Yellow, Disperse Blue 56, Disperse Red 343	[83]
	Cellulose Acetate	Disperse Red 60 and 82	[75]
	Vinylon fibre	Reactive Red 2	[95]
	Cotton	Acid	[96]
	Cotton and cotton/polyester	Basic Red 14, Basic Blue 3, Basic Yellow 24 and 13	[97]
	Polyester/Wool	Disperse Red 54 and 167, Disperse blue 183	[98]
	Polypropylene	Disperse, acid and reactive	[99]

**Table 3:** Studies that used cyclodextrin to graft finishes in textiles.

Fiber	Effect	Active Molecule	Reference
Cotton	Antimicrobial	Octenidine dihydrochloride	[138]
		Silver	[139,140]
		Phenolic compounds	[76,141]
		Ketoconazole	[115]
		ZnO, TiO2 and Ag nanoparticles	[142]
		Miconazole nitrate	[143]
		Triclosan	[144]
	Fragrance, antimicrobial	Essential Oils	[74,145]
	Insect repellent	Cypermethrin and Prallethrin	[146]
	Nocturnal regulation of sleep and antioxidant properties	Melatonin	[77]
	For coetaneous affections	Hydrocortisone acetate	[147]
Polyamide	Perfume, moisturize and UV-protect.	2-ethoxynaphtalene (neroline)	[119]
	Antibiotics	Ciprofloxacin	[148]
Tencel	Sunscreen	Octyl methoxycinnamate	[149]
	Fragrance, antimicrobial and insect repellent	Vanillin, benzoic acid andIodine, *N,N*-diethyl-m-toluamide and dimethyl-phthalate	[44]
Polyester	Antibiotics	Ciprofloxacin	[150]
	Antimicrobial	Curcumin	[151]
		4-tert-butylbenzoic acid	[152]
Wool	Insect repellent	Citronella essential oil	[78]
Cotton and Polyester	Insect repellent	Citronella essential oil	[38]
Cotton, wool and polyester	Fragrance	β-citronellol, camphor, menthol, cis-jasmone and benzyl acetate	[153]

**Table 4:** Use of cyclodextrins as a removal agent in the textile process.

Method	Dye	Reference
Cyclodextrin incorporated into a matrix	Crystal Violet	[162]
	Reactive Black 5	[80]
	Methylene Blue	[174]
	Methyl Orange	[175]
	Safranin O, Brilliant Green and Methylene Blue	[161]
	Methylene Blue and Safranine T	[176]
	Methylene Blue, Acid Blue 113, Methyl Orange and Disperse Red 1	[177]
	Remazol Red 3BS, Remazol Blue RN, Remazol Yellow gelb 3RS 133	[178]
	Methyl Blue	[169]
β-cyclodextrin polymer	Acid Blue 25, Reactive Blue 19, Disperse Blue 3, Basic Blue 3 and Direct Red 81	[81]
	Basic Blue 3, Basic Violet 3 and Basic Violet 10	[170]
	Direct Violent 51, Methyl Orange, And Tropaeolin 000	[179]
	Congo Red and Methylene Blue	[41]
	Evans Blue, Chicago Sky Blue, Benzidine, P- hloroaniline	[180]
	Methylene Blue And Methyl Orange	[42]
	Congo Red, Methylene Blue, Methylene Orange	[156]
	Basic Orange 2, Rhodamine B, Methylene blue trihydrate, and Bisphenol A	[40]
	Methyl orange, Congo Red, Rhodamine B	[181]
	Direct Red 83:1	[171]
